# Supplementary material for: Transcriptomic modulation in response to an intoxication with deltamethrin in a population of Triatoma infestans with low resistance to pyrethroids
Source: PLoS Negl Trop Dis. 2022 Jun 29;16(6):e0010060. doi: 10.1371/journal.pntd.0010060 (PMC9275713; doi:10.1371/journal.pntd.0010060)
Supplement: S1 File — (PDF) [file pntd.0010060.s001.pdf]

S1 File. Summary of the pipeline used for data curation and analysis.

1) **Data preprocessing**: The raw reads from the eight samples were analyzed using Trimmomaticv0.36 as shown in this example command:

```
$ java -jar trimmomatic-0.36.jar PE -threads 20 -phred33 \
sampleX_1.fq.gz sampleX_2.fq.gz \
sampleX.paired.1.fastq.gz sampleX.single.1.fastq.gz \
sampleX.paired.2.fastq.gz sampleX.single.2.fastq.gz \
TRAILING:5 LEADING:5 SLIDINGWINDOW:4:18 \
ILLUMINACLIP:TruSeq3-PE-2.fa:2:30:10 MINLEN:50 2> sampleX.log
```

2) **Transcriptome assembly:** The resulting trimmed and quality-filtered paired-end reads of the eight samples obtained in Step 1 were used to construct a *de novo* transcriptome assembly using the software Trinity v2.10.0 with default parameters. Example command:

```
$ Trinity --seqType fq --max_memory 25G \
--left sampleX.paired.1.fastq.gz,sampleY.paired.1.fastq.gz \
--right sampleX.paired.2.fastq.gz,sampleY.paired.2.fastq.gz \
--CPU 10 > run.log 2>&1 &
```

3) **Prediction of coding sequences:** The resulting *de novo* assembled transcriptome was analyzed with Transdecoder software v5.5.0 to search for coding sequences. A detailed description of the usage of this software is available at https://github.com/TransDecoder/TransDecoder/wiki

   3A) *TransDecoder.LongOrfs* script was used to extract the Open Reading Frames (ORFs) with a product of at least 100 amino acids from the assembled transcriptome.

   3B) The file *longest_orfs.pep* generated in 3A step was used for homology searches (see Methods section from the main text for details).

   3C) Final predictions were obtained using *TransDecoder.Predict* script along with the output from the searches obtained in step 3B.

4) **Sequence clustering:** CD-HIT v4.8.1 in the cd-hit-est mode was run with a sequence identity threshold of 0.95 on the Trandecoder .cds output (`Trinity.fasta.transdecoder.cds`), to obtain the non-redundant CDS database used to perform the following analyzes.

```
$ cd-hit-est -o non-redundant-CDS-database.cds -c 0.95 \
-i Trinity.fasta.transdecoder.cds -p 1 -d 0 -b 3 -T 2
```

5) **Completeness analysis**: BUSCO v4.1.4 software was run against the protein sequences extracted from the STEP 4 resulting database (`non-redundant-CDS-database.cds`), against hemiptera_odb10 database in protein mode.

6) **Transcript quantification:** This step was run using Trinity software *.pl* scripts (v2.10.0), along with Salmon software (a detailed description of the usage of this script is available at https://github.com/trinityrnaseq/trinityrnaseq/wiki/Trinity-Transcript-Quantification)

   6A) The script *`align_and_estimate_abundance.pl`* script was used for preparing the reference database and to map the cleaned and trimmed reads from each library against it.

6B) The *abundance_estimates_to_matrix.pl* script was used to construct the count matrix used to perform the differential expression analysis as described in the Methods section from the manuscript.
