# Supplementary figures and images for: Transcriptomic modulation in response to an intoxication with deltamethrin in a population of Triatoma infestans with low resistance to pyrethroids
Source: PLoS Negl Trop Dis. 2022 Jun 29;16(6):e0010060. doi: 10.1371/journal.pntd.0010060 (PMC9275713; doi:10.1371/journal.pntd.0010060)

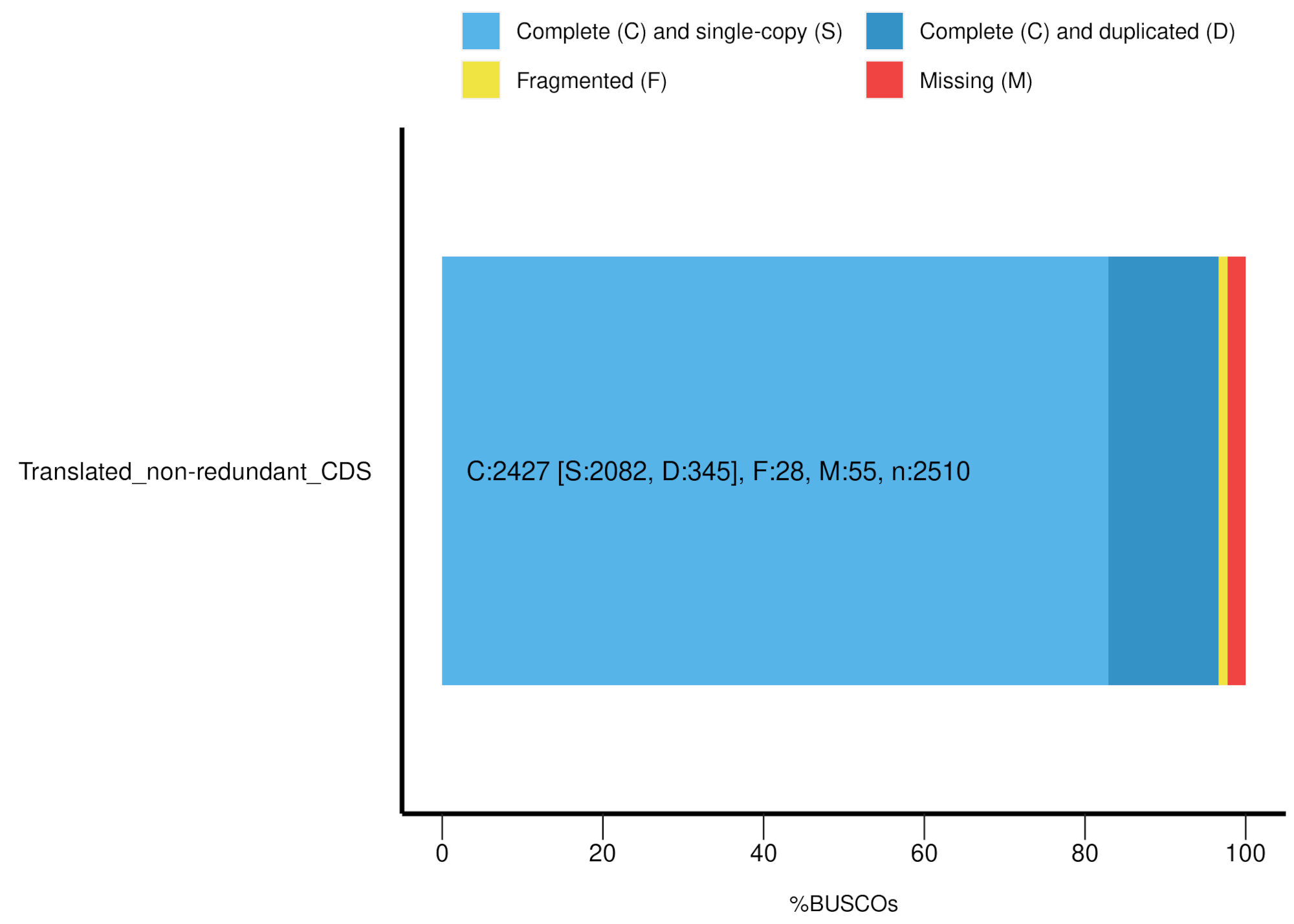

Supplement: S1 Fig — (TIF) [file pntd.0010060.s003.tif]

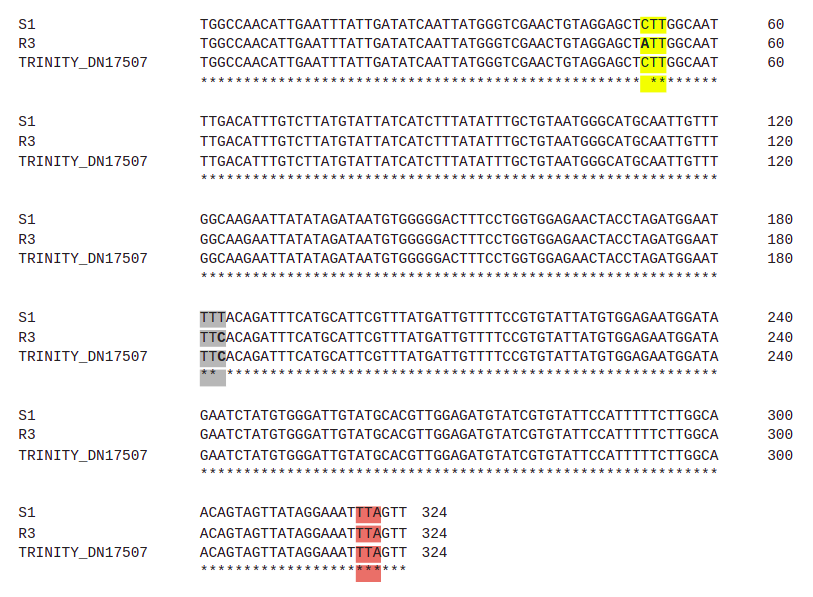

Supplement: S2 Fig — S1: pyrethroid-susceptible; R3: pyrethroid-resistant from Güemes department [9]. The fragment of TRINITY_DN17507 shown here is represented by two sequences in the non-redundant CDS dataset. Nucleotide changes in comparison to S1 are shown in bold. Yellow: Codon where L925I substitution can occur. Red: Codon where L1014F substitution can occur (not present in any of the sequences analyzed here). Grey: Codon with a silent mutation detected in the assembled transcriptome, present in 63% of the mapped reads. Asterisk indicates conserved positions. (TIF) [file pntd.0010060.s004.tif]

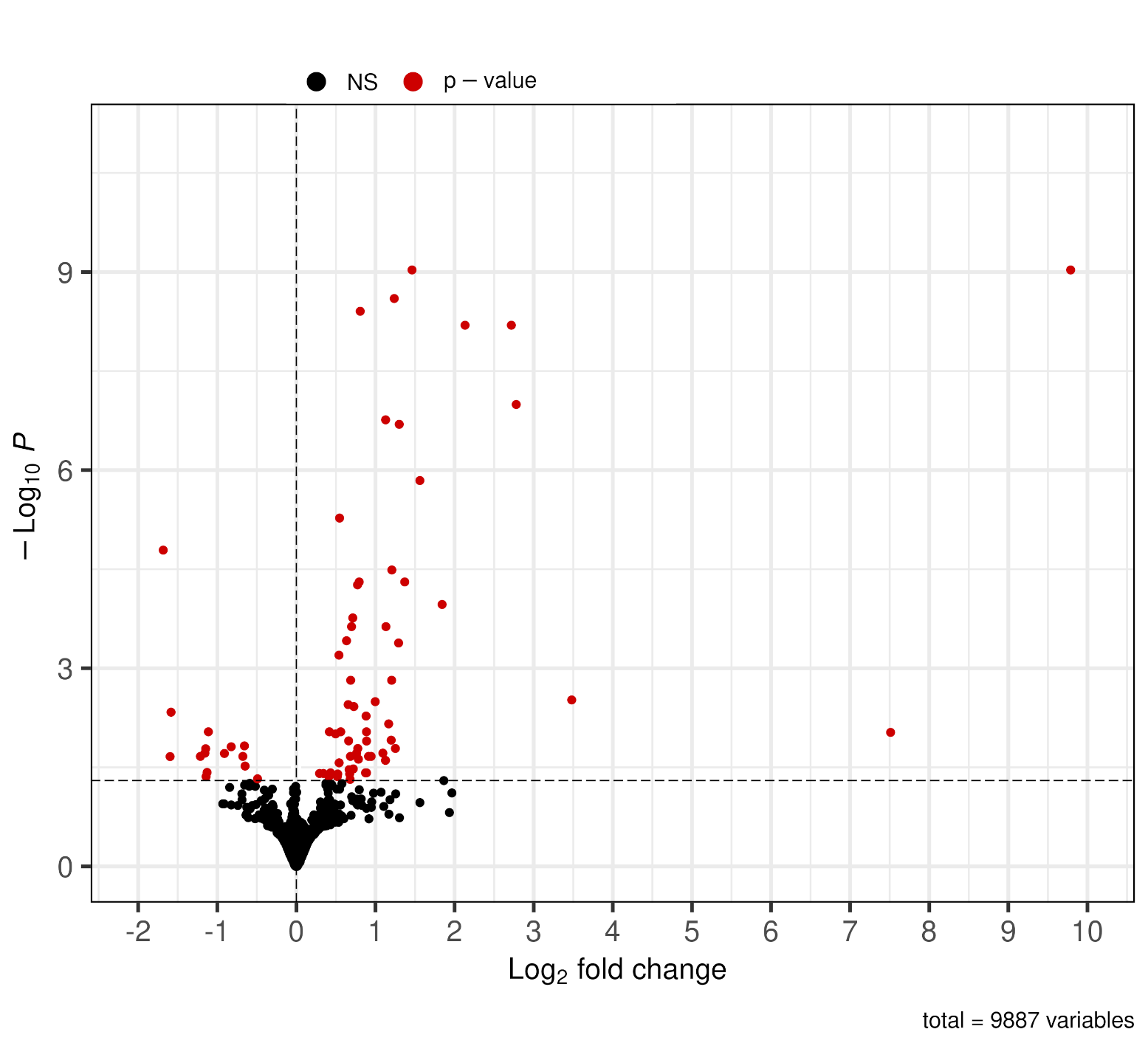

Supplement: S3 Fig — X-axis and y-axis represent log2 fold-change (FC) differences between the compared groups and statistical significance as the negative Log of P-values, respectively. The abundance of 9,887 transcripts (generated after the filtering step) was plotted. Transcripts with p-value (FDR) < 0.05 are indicated with red dots and non-significant transcripts are shown as black dots. (TIF) [file pntd.0010060.s005.tif]
