# Supplementary material for: Transcriptomic modulation in response to an intoxication with deltamethrin in a population of Triatoma infestans with low resistance to pyrethroids
Source: PLoS Negl Trop Dis. 2022 Jun 29;16(6):e0010060. doi: 10.1371/journal.pntd.0010060 (PMC9275713; doi:10.1371/journal.pntd.0010060)
Supplement: Attachment — Submitted filename: Response to reviewers.docx [file pntd.0010060.s006.docx]

Dear Editor:

Thank you for dealing with our manuscript. Present version addressed all the reviewers’ comments, which we believe that really helped to improve the manuscript.

Please find below the detailed response to Reviewers:

**Reviewer #1:** Methods are clearly described, as stated in the comments below, the non-standard pipelines should be available on github or alternatively, the commands used reported in the manuscript.

**Author response:** Thanks to Reviewer 1 for this positive comment. We have included a summary of the pipeline used in the manuscript in a supplementary file (S1 file) in the new version.

**Reviewer #2:** The objectives of the study are clearly articulated. The study design is appropriate and were correctly analysed
No concerns about ethical or regulatory requirements.

**Author response:** Thanks to Reviewer 2 for this comment.

**Reviewer #3:** The study design and the methods used for transcriptomic analysis are in general appropriate and correctly articulated with the objectives. However, I think there are some minor points the authors should address before the manuscript is acceptable for publication.

**Author response:** Thanks to Reviewer 3 for this comment.

-  As the authors recognize that the resistance phenomenon may be very variable depending on the resistance factor being involved, they should not generalise their findings to the whole species. Therefore, the title and other parts of the manuscript should reflect that the study is based on a particular low resistance T. infestans strain.
- Please, check the availability of the transcriptomic data, as searching within the NCBI database shows that the bioproject does not exist.

**Author response:** According to Reviewer 3 suggestion, we have included modifications to indicate that the results from our work could not be generalized for the whole species. Besides, transcriptomic data has now been released by NCBI.
--------------------

<b>Results</b></br></br>
-Does the analysis presented match the analysis plan?</br>
-Are the results clearly and completely presented?</br>
-Are the figures (Tables, Images) of sufficient quality for clarity?</br></br>

**Reviewer #1:** The results are well described and interesting.

Figure 1 - I assume these are counts of some kind? Should specify what exactly this is.
Figure 2/3/6 - I can't read the branch labels at all
Some CSPs were also down regulated in An. gambiae after exposure
evolutive pressure should be evolution pressure
A little more details about the tissue specificity of the ABC family would be interesting for discussion

**Author response:** Thanks to Reviewer 1 for these observations; all of them have been addressed in the revised version of the manuscript. Figure 1 shows a plot of z-scores calculated from the transformed count data; this explanation was added in the figure description. Regarding figures 2/4/6, new versions of them were included to facilitate the visualization of the branch support values: UFBoot values were deleted, and branches that present SH-aLRT values > 80 were marked with a dot.

**Reviewer #2:** Results are clearly and completely presented.
Figures and tables are okay.

**Author response:** Thanks to Reviewer 2 for this comment.

**Reviewer #3**: - In general, the results are clearly and exhaustively presented, especially those related to chemosensory proteins. However, the authors did not validate the differential expression results obtained from RNA-seq by any independent method. qPCR has historically been used to confirm data obtained in transcriptomics studies and despite the debate on whether qPCR validation of transcriptomics data is needed there is a consensus that it is especially useful to corroborate de expression of genes with low fold change values, which is the case of many genes in this study. Thus, I think the authors should carry out a qPCR analysis on some randomly chosen genes using independent cDNA samples of this Triatoma infestans strain in order to validate their present differential expression results. Furthermore, authors rose questions about the lack of differential expression in detoxifying genes such as cytochrome P450, esterases and others as overexpression of these genes has
been reported in previous studies, which led them to speculate that this discrepancy might be related to insect strain being used and exposure time. And this issue can be easily solved with a qPCR analysis of genes coding for detoxifying enzymes in order to corroborate or not the results from RNA-seq.

**Author response:** Thanks for this comment and suggestion. Taking into account the absence of qPCR validation of our RNA-Seq results, we were very conservative with the differential expression analysis in order to minimize the possibility of reporting false positives or negatives, even for those genes with low fold change. First, we removed those genes highly variable and low expressed from our dataset that could generate noise in the analysis. Second, we selected a False Discovery Rate (FDR) <0.05 to identify differentially expressed genes. Third, the *apeglm* method (Approximate Posterior Estimation for Generalized Linear Model) from DESeq2 software (which reduces variance while preserving the true large effect sizes) was used in our analysis, improving fold-change estimation (see reference 1 below). It is important to mention that we found that only 10% of our DE set showed a low fold change (log_2_ FC lower than 0.5).

Besides, results and discussion of the manuscript are mainly focused in biological processes and gene families rather than in individual genes, providing a general vision of the pyrethroid effects at molecular level. Nevertheless, qPCR validation will be necessary in the case of performing functional genetic studies with a particular gene that showed differential expression in our work. For this, we have included a sentence about this point in the edited version of the manuscript.

Last, it is important to mention that RNA-Seq is a highly sensitive and reproducible technique. It is now widely accepted that this technique correlates extremely well with single gene RT-qPCR validations, and RNA-Seq is less biased and imprecise than RT-qPCR (see for example references 2-5 below).

For all these reasons, we consider that qPCR validations of DE genes will not provide new or significant information to this work. Moreover, RNA-Seq represents an excellent opportunity to study the expression of large gene families (as is the case of CYPs, CCEs and GSTs) in the same experiment, which would be impossible to address with other methods such as RT-qPCR. For this reason, the validation of the absence of differential expression of detoxifying genes of this species would be extremely expensive and time-consuming to use to validate a robust technique as RNA-Seq.

1) Zhu, A., Ibrahim, J. G., & Love, M. I. (2019). Heavy-tailed prior distributions for sequence count data: removing the noise and preserving large differences. Bioinformatics, 35(12), 2084-2092.

2) Griffith M, Griffith OL, Mwenifumbo J, Goya R, Morrissy a S, et al. (2010) Alternative expression analysis by RNA sequencing. Nat Methods 7: 843–847. Doi:10.1038/nmeth.1503.

3) Asmann YW, Klee EW, Thompson EA, Perez E a, Middha S, et al. (2009) 3’ tag digital gene expression profiling of human brain and universal reference RNA using Illumina Genome Analyzer. BMC Genomics 10: 531. Doi:10.1186/1471-2164-10-531.

4) Wu AR, Neff NF, Kalisky T, Dalerba P, Treutlein B, et al. (2014) Quantitative assessment of single-cell RNA-sequencing methods. Nat Methods 11: 41–46. Doi:10.1038/nmeth.2694.

5) Shi Y, He M (2014) Differential gene expression identified by RNA-Seq and qPCR in two sizes of pearl oyster (Pinctada fucata). Gene 538: 313–322. Doi:10.1016/j.gene.2014.01.031.

- please include in Fig. 1 legends a brief explanation of what does the dendrogram on the left mean.

**Author response**: Thanks for the suggestion. The legends from figures 1 and 7 were modified adding an explanation of the meaning of dendrograms on each heatmap. Moreover, these explanations were also improved in the Methods section of the manuscript.
--------------------

<b>Conclusions</b></br></br>
-Are the conclusions supported by the data presented?</br>
-Are the limitations of analysis clearly described?</br>
-Do the authors discuss how these data can be helpful to advance our understanding of the topic under study?</br>
-Is public health relevance addressed?</br></br>

**Reviewer #1:** See above

**Reviewer #2**: The conclusions are  supported by the data presented.

**Reviewer #3:** In general, the conclusions are supported by the data presented and the relevance of the genomic data is correctly discussed. May be the authors could discuss the limitations of the study related to the insect strain used for the analysis (why they chose to use a single low resistance strain instead of two or three strains with different resistance levels), the time the insects were processed after insecticide application (why they chose a 4 h exposure time instead of a longer one).

**Author response:** In agreement to Reviewer 3, this discussion has been addressed in the revised manuscript.
--------------------

<b>Editorial and Data Presentation Modifications?</b></br><br/>
Use  this section for editorial suggestions as well as relatively minor modifications of existing data that would enhance clarity. If the only modifications needed are minor and/or editorial, you may wish to recommend “Minor Revision” or “Accept”.

**Reviewer #1:** Intro - minor corrections
The line ending in dissimilar success needs rephrasing
'its extension', I guess that should be 'its extent', also a citation would be good
The ABC superfamily, which is much less studied...have been associated...
Would be nice to be a bit more descriptive in the line starting 'we also performed a comprehensive...'

Reviewer #2: (No Response)

Reviewer #3: (No Response)

**Author response:** We thank reviewer 1 for these corrections, which have been addressed in the manuscript.
--------------------

<b>Summary and General Comments</b></br></br>
Use  this section to provide overall comments, discuss strengths/weaknesses of the study, novelty, significance, general execution and scholarship. You may also include additional comments for the author, including concerns about dual publication, research ethics, or publication ethics. If requesting major revision, please articulate the new experiments that are needed.

**Reviewer #1:** This study utilises a triatoma bug with low levels of insecticide resistance from Argentina to characterise the impact of pyrethroid exposure on the transcriptome 4hours after application. The authors characterise chemosensory proteins and ABC transporters due to overexpression and prior role in resistance. Overall, the paper is very good, easy to follow and interesting. The authors do a very good job of explaining the results and linking them to published literature on pyrethroid resistance. I recommend this paper is publishable pending the described changes. I congratulate the authors on a nice paper of an understudied vector.


Is there a github with the .pl scripts used? It would also be beneficial to have the command used for the curation of the data as this doesn't follow a standard pipeline.

Further detail is needed about the resistance level, it is hard to know what 'low' resistance is - there needs to be some form of reference here.

33321 transcripts seems like a very high number - how many of these are predicted to be splice variants of the same gene? I would expect somewhere in the region of 13k

Table 4 is really hard to follow - I know it is a lot of work, but it needs some kind of reformating to make it more readable. Some of the main points are lost.

**Author response:** Thanks to Reviewer 1 for these comments. All the observations have been addressed in the revised manuscript:

- The .pl scripts used in this work are not custom and were obtained from the references cited in the manuscript. However, to clarify the steps of the analysis, a summary of the pipeline was added as a supplementary file.

- We agree that more than 30k transcripts identified in our database is a very high number. For example, the consensus gene prediction from *Rhodnius prolixus*, a triatomine insect with an annotated genome, contains 15,456 protein-coding genes (6). However, getting a high number of transcripts is not an unusual result in Trinity *de novo* assembly studies (see reference 7 below). For this reason, we have tried to get closer to obtaining a representative transcript for each gene. First, we decided to use only the predicted coding sequences for more than 100 amino acids, going from 431,511 transcripts to 63,890 predicted CDS (using Transdecoder). Second, transcripts with more than 95% of nucleotide identity were grouped using CD-HIT, going from 63,890 to 33,321 sequences (the non-redundant CDS-database). In the differential expression analysis step, we performed an extra filtering step, and eliminated those genes with highly variable and/low expression getting a final dataset of 9,887 transcripts to perform the differential expression analysis. A similar scenario was found, for example, in a recent work published by Tanaka *et al*. The authors analyzed the antennal transcriptome of a beetle, finding a total of 23,840 contigs after a clustering step (98%) (see reference 8 below).

Despite all these filtering steps, we can not rule out if some CDS represent isoforms/splicing variants, fragments of the same gene, or if they are actually encoded in different genes. An annotated genome of *T. infestans* would be needed to assess this point. For this reason, we have been cautious and discussed this throughout the manuscript, especially when comparing the number of representatives found for each family in comparison with other insects.

6) Mesquita RD, Vionette-Amaral RJ, Lowenberger C, Rivera-Pomar R, Monteiro FA, Minx P, et al. Genome of Rhodnius prolixus, an insect vector of Chagas disease, reveals unique adaptations to hematophagy and parasite infection. Proc Natl Acad Sci. 2015;112: 14936–14941. doi:10.1073/pnas.1506226112

7) https://github.com/trinityrnaseq/trinityrnaseq/wiki/There-are-too-many-transcripts!-What-do-I-do%3F

8) Tanaka K, Shimomura K, Hosoi A, Sato Y, Oikawa Y, Seino Y, et al. Antennal transcriptome analysis of chemosensory genes in the cowpea beetle, Callosobruchus maculatus (F.). PLoS One. 2022;17: e0262817. doi:10.1371/journal.pone.0262817

- Table 4 in the supplementary file S2 was reformatted for a better visualization of results. Moreover, a detailed explanation of each field was added in the table description, and additional details were added in the Methods section of the manuscript.

**Reviewer #2**: Comments to the manuscript PNTD-D-21-01722 intended as an research article in PLOS Neglected Tropical Diseases entitled “Transcriptomic modulation in response to an intoxication with deltamethrin in Triatoma infestans, vector of Chagas disease” by Traverso L, Estivalis JML, Fernandes GR, Fronza G, Lobbia P, Cueto GM, Ons S.
They present a very interesting manuscript investigating the transcriptomic alterations caused by the synthetic pyrethroid deltamethrin in a low pyrethroid-resistant population of Triatoma infestans. The methods used and the analyses performed seem appropriate and solid. The manuscript has a good and comprehensive introduction to the topic and the results are clear and concise presented.

The work presented is to a large extend descriptive reporting of data, which is the nature of an initial transcriptome paper. The authors related their findings nicely to previous findings in other insects with pyrethroids or other insecticides. The authors should be praised for having a focus on e.g. chemosensory proteins and not just CYPs or GSTs, but it would be very interesting with some hypotheses or interesting questions on CSPs to enlighten the discussion and add more intellectual value to the manuscript.
The study is an important stepping stone to focus on the insecticide detoxification in Triatoma infestans.

**Author response:** Thanks to Reviewer 2 for these comments. In agreement, we have enriched the discussion on the probable mechanisms of the CSP family.

**Reviewer #3**: The manuscript by Traverso et al. presents a transcriptomics analysis of Triatoma infestans first-stage nymphs from a low pyrethroid-resistant focus 4 hours after being exposed to a sublethal dose of deltamethrin. Whilst the selection of a single population from a region with a wide range of resistance levels and the short time of insecticide exposure may limit the findings of the study, relevant information on the differential expression of some genes has been obtained, especially regarding the relationship between chemosensory proteins and insecticide resistance. And most importantly, the study contributes to increasing the genomic information of this major vector of Chagas disease as its genome is not currently available.

**Author response:** Thanks to Reviewer 3 for these comments. In agreement, we discussed the limitations of the work in the revised manuscript.
